# An Interactive Smartphone App, Nenne Navi, for Improving Children’s Sleep: Pilot Usability Study

**DOI:** 10.2196/22102

**Published:** 2020-12-01

**Authors:** Arika Yoshizaki, Ikuko Mohri, Tomoka Yamamoto, Ai Shirota, Shiho Okada, Emi Murata, Kyoko Hoshino, Kumi Kato-Nishimura, Shigeyuki Matsuzawa, Takafumi Kato, Masako Taniike

**Affiliations:** 1 Molecular Research Center for Children's Mental Development United Graduate School of Child Development Osaka University Suita, Osaka Japan; 2 United Graduate School of Child Development Osaka University Suita, Osaka Japan; 3 Department of Oral Physiology Graduate School of Dentistry Osaka University Suita, Osaka Japan; 4 Segawa Memorial Neurological Clinic for Children Chiyoda, Tokyo Japan; 5 Ota Memorial Sleep Center Kawasaki, Kanagawa Japan

**Keywords:** infant sleep, app, mHealth, behavioral intervention, sleep health, PDCA cycle

## Abstract

**Background:**

Healthy sleep is important not only for physical health but also for brain development in children. Several reports have revealed that Japanese adults and children have later bedtimes and shorter sleep durations compared with those in other countries, possibly because of Japanese culture and lifestyles. Therefore, an intervention tool that is suitable to the Japanese sociocultural environment is urgently needed to improve children’s sleep problems in their early years.

**Objective:**

To provide appropriate sleep health literacy to caregivers and change their parenting behavior, we developed a smartphone app that allows reciprocal interaction between caregivers and pediatric sleep experts. This paper describes a preliminary study to examine the app’s basic design and functions and to establish its acceptability and usability in a small sample.

**Methods:**

A total of 10 caregivers and 10 infants (aged 18-28 months; 4/10, 40% boys) living in Japan participated in the study. At the start of the trial, the e-learning content regarding sleep health literacy was delivered via a smartphone. Thereafter, caregivers manually inputted recorded data about their own and their infant’s sleep habits for 8 consecutive days per month for 2 months. After pediatric sleep experts retrieved this information from the Osaka University server, they specified the problems and provided multiple sleep habit improvement suggestions to caregivers. Caregivers then selected one of the feasible pieces of advice to practice and reported their child’s sleep-related behaviors via the app. Actigraphy was used to monitor children’s sleep behaviors objectively. The concordance between the information provided by caregivers and the actigraphy data was assessed. The acceptability and usability of the app were evaluated using self-report questionnaires completed by caregivers; qualitative feedback was obtained via semistructured interviews after the intervention.

**Results:**

There was no significant difference between the information provided by the caregivers and the actigraphy data for bedtimes and wake-up times (*P*=.13 to *P*=.97). However, there was a difference between the actigraphy data and the caregivers’ reports of nighttime sleep duration and nighttime awakenings (*P*<.001 each), similar to prior findings. User feedback showed that 6 and 5 of the 10 caregivers rated the app easy to understand and easy to continue to use, respectively. Additionally, 6 of the 10 caregivers rated the app’s operativity as satisfactory. Although this was a short-term trial, children’s sleep habits, caregivers’ sleep health consciousness, and parenting behaviors improved to some extent.

**Conclusions:**

The present findings suggest that the app can easily be used and is acceptable by Japanese caregivers. Given the user feedback, the app has the potential to improve children’s sleep habits by sending individualized advice that fits families’ backgrounds and home lives. Further studies are needed to confirm the efficacy of the app and facilitate social implementation.

## Introduction

### Sleep Problems in Childhood

Sleep problems in children is one of the most common caregiver concerns, regardless of cultural origins [1-3]. Previous research has shown that a good quantity of high-quality sleep during early childhood is crucial for later development [4]. For example, shorter sleep duration during the first 3 years of life has been identified as a risk factor for hyperactivity and lower cognitive performance in later childhood [5,6]. Additionally, children’s sleep problems are associated with parental stress [7] and maternal depressive symptoms [8].

Sleep specialists, including pediatricians and psychologists, have long been responsible for educating caregivers of infants with sleep problems, such as sleep-onset insomnia and night waking, in pediatric sleep clinics.

### Sleep Problems in Families and Impact on Children’s Sleep

In a previous study comparing sleep habits and problems in 16 countries [9], Japanese children were found to have the shortest sleep times. More recently, using a validated questionnaire developed for screening children’s sleep problems and the sleep-related lifestyles in Japanese families [10-12], researchers found that the mean sleep time in Japanese preschoolers was as short as 9.7 hours. Interestingly, 55% of caregivers whose children slept less than 9 hours believed that their children had good sleep [13]. Furthermore, children’s sleep time was found to be strongly affected by their families’ lifestyles, including watching television near bedtime and being out after 8:00 PM.

Several reports revealed that Japanese adults also have shorter sleep times [14,15], probably due to Japanese cultural notions that value hard work over sleep. These findings highlight both children’s shorter sleep times and caregivers’ low awareness of the issues involved. Given the known consequences of insufficient sleep for children and parents, there is an urgent need to develop an intervention for improving caregivers’ lifestyles in order to positively impact children’s sleep habits in the early years while also considering sociocultural factors, such as increasing dual-income and nuclear families, and Japanese sleep-related cultural factors, such as co-sleeping habits.

In Japan, parenting guidance for caregivers has traditionally been provided on a face-to-face basis during children’s regular checkups at regional public health centers [16]. However, sleep problems are often overlooked at these checkups because of a paucity of sleep specialists. Even if these problems are addressed, it is difficult for caregivers to visit public health centers for repeated consultations and follow-up, as double-income families now account for the majority of the Japanese population. It is difficult to deliver appropriate sleep hygiene information to caregivers because of their lack of adequate sleep literacy, poor motivation to make behavioral changes, or difficulty identifying improvements that they can apply in their own homes.

Sadeh et al [2] suggested a transactional model that emphasizes the ongoing bidirectional links between parenting and infant sleep, such as parental, infant, intrinsic, and sociocultural factors, among others. Besides the changes in Japanese families’ lifestyles, children’s sleep environments have also been worsening considerably in recent years. Smartphones are very popular, and infants’ exposure to such media is a major issue affecting sleep hygiene [17,18].

Some studies have examined online or mobile health education or interventions for infants’ sleep [19-21]. However, interactive interventions could be more effective, given the variance in families’ socioeconomic status, bedroom environments, and caregiver beliefs regarding sleep. Sviggum et al [22] showed that early, customized guidance for caregivers that focuses on revealing and acknowledging their experiences of sleep problems in their children is essential in helping caregivers cope with such challenges. Recent findings suggest that parental factors both predict and are predicted by behavioral interventions for infant sleep problems [23]. To improve children’s sleep habits effectively, individualized care is desirable, such as care supported by an interactive plan-do-check-act (PDCA) cycle for caregivers.

Against this background, we developed a smartphone app that facilitates interaction between caregivers and pediatric sleep experts to improve infants’ sleep habits in Japan. Using the app, caregivers reported their children’s sleep and sleep-related schedules for 8 consecutive days in a month, and the sleep team provided various types of advice after analyzing the children’s sleep habits. Educational content was also delivered via smartphone. Recent reviews have highlighted the need for further evaluation of mobile health interventions in children [24,25].

The aim of the present study was to describe the app’s developmental design; check the system’s operation, acceptability, and usability; and determine the overall potential of the app to change infants’ sleep habits or parental cognition and behavior via user feedback in a small trial.

### A Priori Hypotheses

We hypothesize that the app would work well and with little discomfort for Japanese caregivers, since its concept and design required minimal effort. We also predict that the caregivers’ input information regarding sleep-related habits will be reliable compared with the data from the actigraph.

## Methods

### Ethics Approval and Consent to Participate

This study was approved by the Osaka University Clinical Research Review Committee (CRB5180007) on January 23, 2017, prior to the start of the study. All study procedures were conducted in accordance with the ethical standards of the Declaration of Helsinki. Written consent was obtained from all participants on an individual basis. All participants received a coupon for books valued at US $20 upon completion of the trial.

### Participants

The study targeted only caregivers with infants aged 18 to 36 months, as the app was designed for this age group. A total of 10 participants and 10 infants were recruited from the university community over a 2-week period using a bulletin board in the nursery. The inclusion criteria were as follows: (1) fluency in Japanese, (2) possession of a mobile device (iOS or Android) with internet access, and (3) willingness to install the Nenne Navi app on the mobile device. Supported devices included the iPhone, iPad, and iPod touch with iOS 8.0 or later and Android devices with Android OS 4.3 or later.

The ratio of iOS (all iPhones) to Android devices was 6:4. The mean age of the infants was 22.6 months (range 18-28 months; 4/10, 40% boys). The mean age of the caregivers (8 mothers and 2 fathers) was 36.1 years (range 31-41 years), and the ratio of family care (children at home) to professional childcare was 2:8. A total of 7 caregivers worked at Osaka University (4 medical professionals, 1 medical research assistant, and 2 office workers), and 1 caregiver was a graduate student. One caregiver was a homemaker. One caregiver (medical professional) was from another Asian country, had lived in Japan for several years, and had sufficient Japanese communication skills for participation.

### Measures

#### App: Nenne Navi

**Figure 1:**
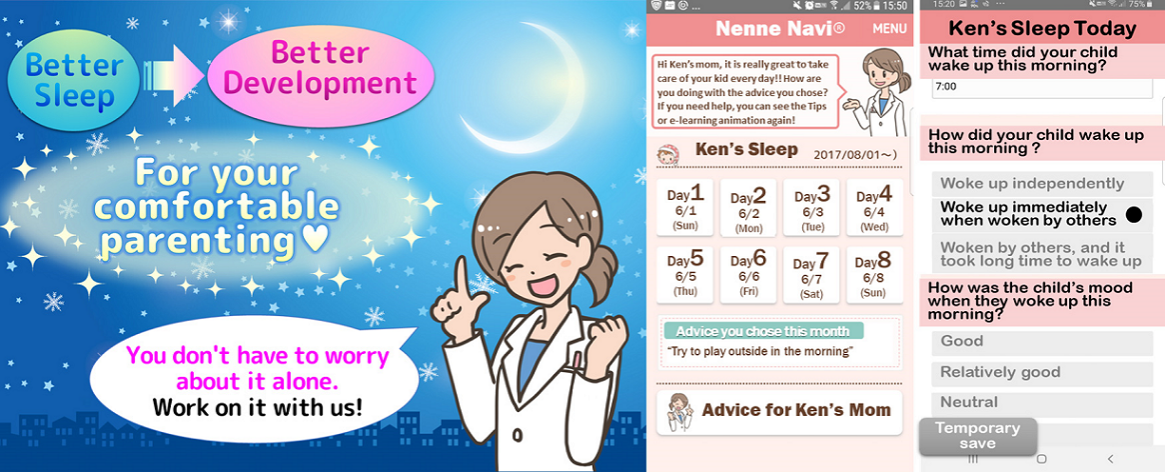
Nenne Navi screenshots, including the page used to enter lifestyle information and provide users with messages and advice from pediatric sleep experts and the page for data input.

The app, Nenne Navi, was developed by pediatric sleep experts at the pediatric sleep clinic at Osaka University Hospital. The system design was outsourced to a domestic information technology company. The Japanese word *nenne* means “sleep.” The e-learning content regarding sleep health education was delivered via narrated animations. The narrated animation lasted for 3 minutes and included the following: (1) tips for better ways to spend time during the day to ensure good sleep and (2) tips on how to spend time in the evening and nighttime to ensure good sleep. In the animation, a professional character describes the daily activities of 2 boys (a good sleeper, Taro, and a poor sleeper, Jiro; Figure 1).

**Figure 2:**
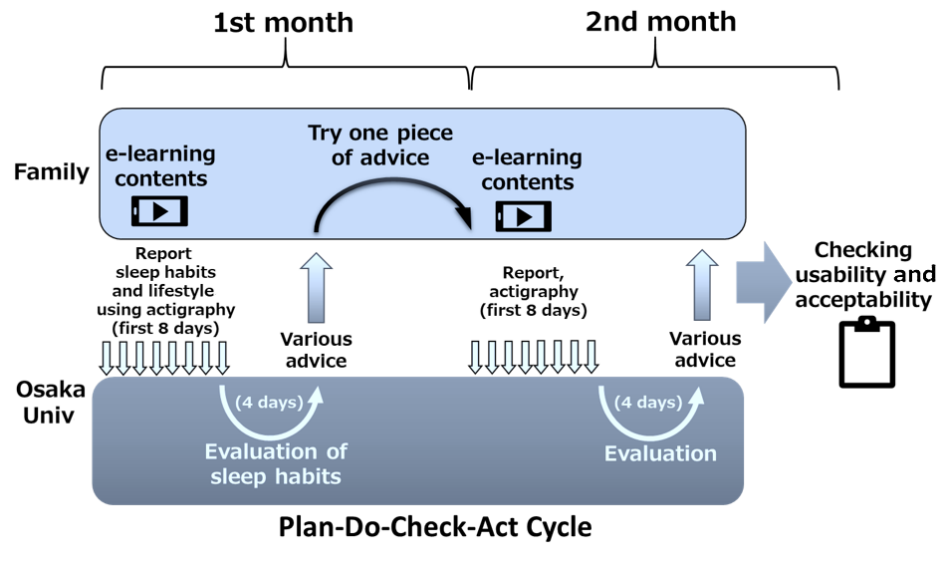
Plan-do-check-act cycle to improve children's sleep habits via the interactive app Nenne Navi. Univ: university.

The caregivers’ input information regarding sleep-related habits included waking time, nap time, bedtime, screen time, outdoor activity time, bathing time, dinner time, how time was spent before retiring to bed, and sleep latency (see Multimedia Appendix 1 for details) for their infants for 8 consecutive days in a month (Figure 1). They also inputted information about their own sleep and wake times, as well as their stress levels and feelings about parental care. Participants continued the PDCA cycle shown in Figure 2.

All the input data were sent to the Osaka University virtual server hosting service developed at Cybermedia Center, Osaka University, which is equipped with network security measures such as access restrictions, encrypted communication, monitoring of unauthorized access, and backup to withstand cyber attacks. The system configuration was as follows: (1) Ubuntu 18+ Apache 2.7, (2) PHP 7, and (3) PostgreSQL 9.

The registration number of the trademark (606435 Nenne Navi, Osaka University; Taniike M, Mohri I, Aoi Y, Yoshizaki A) was registered on July 20, 2018. The patent application number was 2017-202916.

#### Strategy for Intervention: Individualized Small Steps, Autonomous Choice of Behavioral Experiment, and Encouragement to Support Motivation

The pediatric sleep expert team consisted of 3 pediatricians and 2 psychologists who analyzed the information entered by the caregivers and sent various types of practical advice to each caregiver. The app was designed to set personalized goals in accordance with individual users’ home lives; for example, the app sent personalized advice such as “Try to have dinner before 8:00 PM” instead of “Try to have dinner earlier” to deliver specific and optimal goals to caregivers in small steps, based on behavioral therapy concepts. The app did not intend to instruct caregivers to obey the advice. Rather, it was designed to send various pieces of advice (suggestions), from which caregivers could choose one suggestion. We expected that if caregivers chose one suggestion, thinking they would try it for a while without much effort, they would try the app and follow the advice willingly.

Advice categories, which comprised approximately 60 suggestions, included how to spend time before going to bed, how to spend time in the bedroom, establishing a regular sleep rhythm, increasing daytime activity, fixing nap schedules, and fixing dinner and bathing times. Participants were asked to indicate, using the app, which suggestion they chose to try so that the pediatric sleep experts could check their degree of compliance with the advice (Figure 2). At the same time, a feedback message was sent to each caregiver (approximately 200-300 letters in Japanese) through the app. We intended to provide positive feedback on the improvement of lifestyle habits compared with the previous month, empowering caregivers and supporting their motivation.

#### Actigraphy

To collect objective data regarding children’s sleep and check the accuracy of the data input by caregivers, the children’s activity levels were measured using an actigraph (MTB-220; Acos Co Ltd) during the data input period (from days 1 to 8 in the first and second months). The actigraph used was modified to be connectable to the smartphone via Bluetooth instead of FeliCa. This actigraph is a small and light (weight of 9 g) coin-shaped device (external dimensions of 27 mm in diameter and 9.8 mm in depth, including clip) that records amount of physical activity by using an internal 3-axis accelerometer. Every 0.125 seconds, the number of times that acceleration exceeds a reference value is summed, and the value is recorded as the activity value over 2-minute bins. The activity intensity is calculated from the activity value as a value from 0 to 63 (64 levels). An activity intensity of 0 means the participant did not move, and larger values indicate higher levels of activity. The caregivers could attach the actigraph to their infant’s clothes with the clip on the back of the device. Caregivers were asked to press the relevant button on the app to send the actigraph data to the Osaka University server.

#### Questionnaires and Interviews

After the trial, a questionnaire was administered and semistructured interviews regarding the usability and operability of the app were conducted individually to collect information for system improvement. The questionnaire consisted of the following items: ease of continuation of the app, easiness of understanding the app, changes in the child’s sleep habits after using the app, changes in the child’s daytime behavior after using the app, and changes in the easiness of putting the child to sleep after using the app. Responses were provided using 5-point scales (5=satisfied, 4=moderately satisfied, 3=neutral, 2=moderately dissatisfied, 1=dissatisfied) and free-text comments. In the interviews, participants were also asked what motivated their continued use, their impressions of the e-learning content and advice, and how the app system could be improved. To check the feasibility of the app, we asked participants to report that if they had followed the advice.

### Data Analysis

Actigraphy data were sent to the Osaka University server described above and was read by the program Sleep Sign Act (Kissei Comtec Co). After the extraction of CSV data, we determined the wake and sleep times manually using the method described by Nakasaki et al [26]. Briefly, a five-dimensional linear model was hypothesized that uses activity intensity at an evaluation epoch, as well as at two epochs before and two epochs after (total of 10 minutes). Using the activity intensity at 4 minutes and 2 minutes before the evaluation epoch, at the evaluation epoch, and at 2 minutes and 4 minutes after the epoch (*x*_–2_, *x*_–1_, *x*, *x*_+1_, *x*_+2_), each with a weighting coefficient (α_–2_, α_–1_, α, α_+1_, α_+2_), we can determine *z*:

*z* = (0.24669)*x*_–2_ + (0.2562)*x*_–1_ + (0.408771)*x* + (0.155046)*x*_+1_ + (0.136728)*x*_+2_

Here, *z*≥1 denotes wake and *z*<1 denotes sleep.

To compare the number of caregiver-reported nighttime awakenings (defined as those lasting for more than roughly 5 minutes) in the app with those reported by the actigraphy device, we extracted the number of events of wakefulness for 6 minutes or more from the actigraphy data. For the data entered via the app, nighttime sleep was calculated by subtracting sleep latency from the duration of time from bedtime to wake-up. For each value relating to sleep habits and sleep-related lifestyles, the mean, standard deviation, and basic statistics of each participant were calculated for both the app data and the actigraphy data.

To check the reliability of the data caregivers entered via the app, a 2-tailed paired-samples *t* test was used to compare the differences between the actigraphy data and the data entered by caregivers via the app to record children’s sleep habits (ie, bedtime, wake-up time, nighttime sleep, and waking at night). The differences between sleep time duration, mean bedtime, and night awakenings for the first and second months were also assessed (using 2-tailed paired *t* tests). Analyses of these sleep habits were conducted separately for weekdays and weekends, since there could be differences in lifestyle, considering most of the caregivers worked on weekdays. To calculate the correlation value between data entered by the caregivers and actigraphy data for sleep-wake rhythms, the Pearson correlation coefficient was used. Data analysis was performed using IBM SPSS Statistics (v 26.0; IBM Corp).

## Results

### Safety in Use

All 10 caregivers completed the 2-month trial use of the app. No major problems, such as connection errors, were reported. One caregiver discontinued the use of the actigraph because of their fear of accidental ingestion. One participant was excluded from the data analysis for sleep habits because the child was using medication that could have affected sleep during the second month of the study period.

### Usability of Nenne Navi

**Figure 3:**
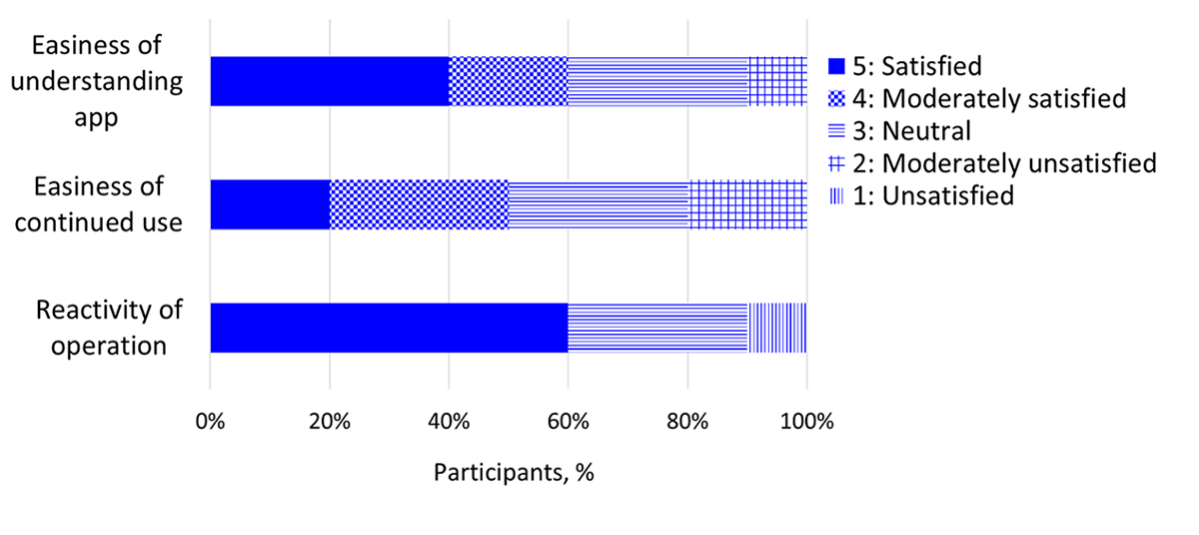
Participants' feedback regarding the usability of the app.

The results of the questionnaire regarding usability and operability showed that only 1 out of 10 caregivers rated the app as unsatisfactory; 6 and 5 of the 10 caregivers rated it as easy to understand and easy to continue to use, respectively. In addition, 6 out of 10 caregivers indicated that they were satisfied in the reactivity of the app’s operation (Figure 3). One caregiver who was unsatisfied with the responsiveness of the app’s operation reported that the screen froze repeatedly; however, the caregiver was able to continue to use the app throughout the trial after restarting the smartphone once. The results of the interviews with caregivers informed functional improvements, such as the addition of a button to save data temporarily.

### Feasibility of Interactive Advice

The advice sent to caregivers by the pediatric sleep expert team consisted of various types of advice concerning children’s daily lives, including increasing daytime activities, controlling media exposure, changing nap schedules, and changing ways to spend time at bedtime. When providing advice to caregivers, the expert team presented goals as clearly as possible (eg, “When your child has a nap, try to wake them before 3:00 PM”). The rate at which caregivers attempted to follow advice (ie, were able to perform advised behavior) was 76.8%.

### Reliability of the Data Entered on the App

**Figure 4:**
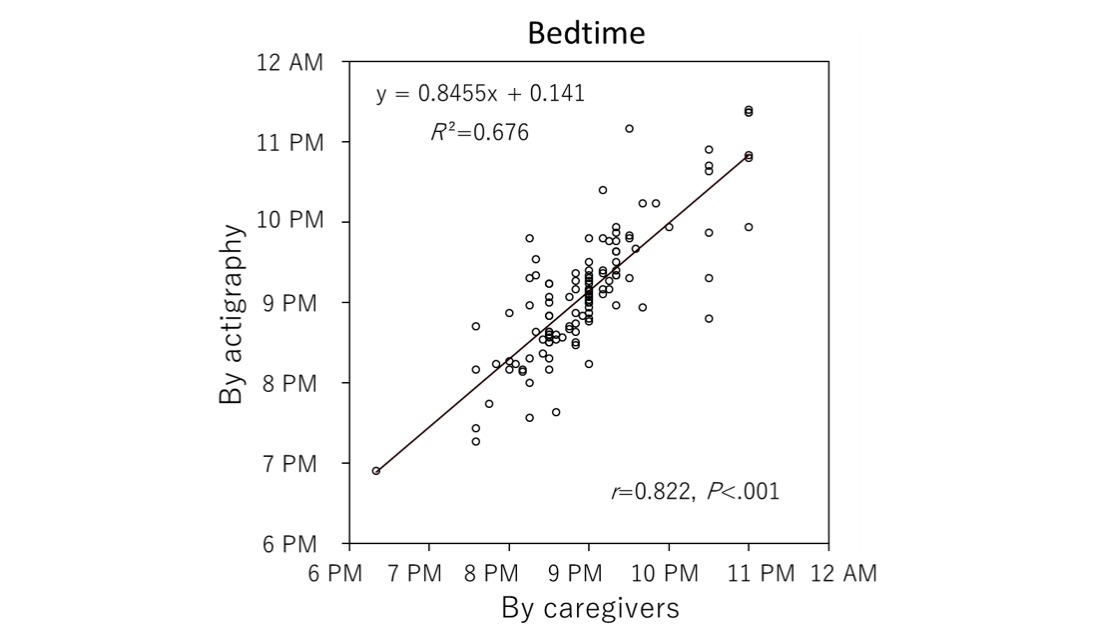
Scatter diagram for the entered data and actigraphy data for bedtimes.

**Figure 5:**
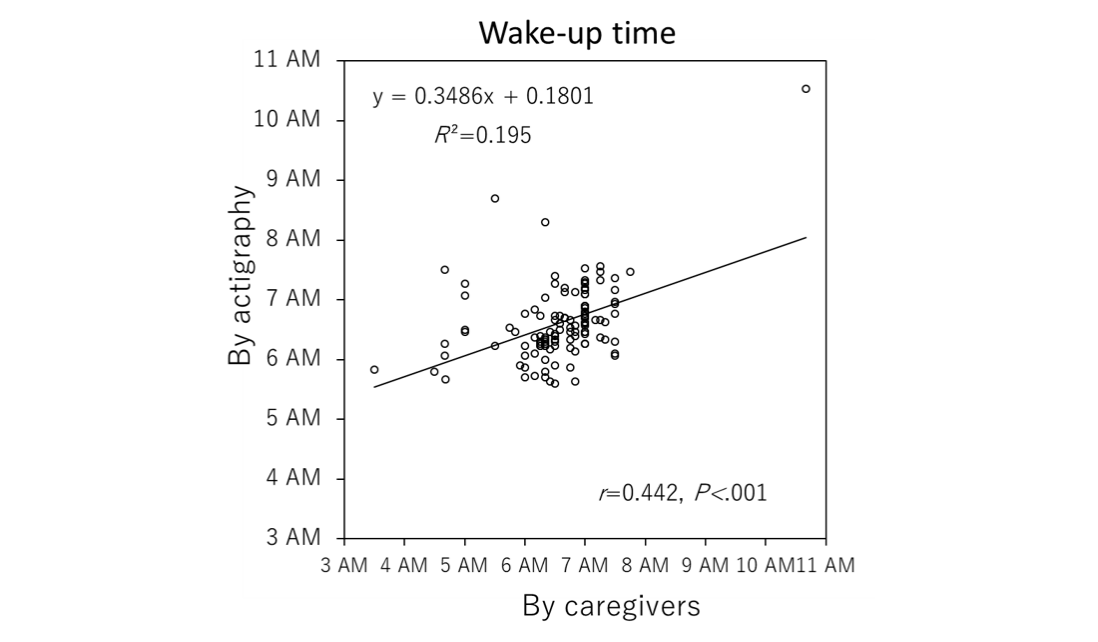
Scatter diagram for the entered data and actigraphy data for wake-up times.

To check the reliability of the data entered by the caregivers, we analyzed the difference between entered data and actigraphy data. As shown in Table 1, there was no significant difference between the data entered by the caregivers and the actigraphy data for bedtime and wake-up time (Figures 4 and 5). However, although the caregivers correctly reported the bedtime of their children (*R*^2^=0.676; *r*=0.822; *P*<.001), the reported wake-up time was less precise than the bedtime (*R*^2^=0.195; *r*=0.442; *P*<.001). In addition, the duration of children’s sleep was found to be significantly shorter in the actigraphy data (approximately 2 hours on average), as the number of night awakenings was significantly higher than that reported by caregivers.

**Table 1:** Children’s sleep habits according to actigraphy and entered data by caregivers.

Measure and month		Participants, n	Actigraphy, mean (SD)	Entered, mean (SD)	*t* test (*df*)	*P* value
**Bedtime**						
	1	10	9:05 PM (0:37)	8:58 PM (0:36)	1.62 (9)	.14
	2	9	9:07 PM (0:42)	8:58 PM (0:43)	1.70 (8)	.13
**Wake-up time**						
	1	10	6:30 AM (0:19)	6:29 AM (0:44)	0.08 (9)	.94
	2	9	6:35 AM (0:31)	6:35 AM (0:38)	–0.04 (8)	.97
**Nighttime sleep (min)**						
	1	10	432 (68.61)	548 (72.88)	–7.06 (9)	<.001
	2	9	433 (62.80)	556 (78.48)	–8.20 (8)	<.001
**Nighttime awakenings (times)**						
	1	10	9.02 (2.78)	0.41 (0.67)	10.13 (9)	<.001
	2	9	8.83 (2.73)	0.47 (0.55)	9.56 (8)	<.001

### Caregivers’ Perceptions of the Effectiveness of the App

According to the results of the interviews conducted after the trial, positive changes were reported in all cases, and no caregivers reported worse sleep habits or childcare burden with the use of the app. The caregivers’ reports regarding the efficacy of the app are shown in Table 2. Overall, the interactive function of the app was evaluated positively by caregivers, as shown in statements such as “It was great that I could receive advice that fit my infant and family.” In addition to the positive feedback, infants’ sleep time was longer on weekends (paired *t* test, *P*=.02), and bathing was finished ahead of schedule on the weekends, but the result was not significant (Multimedia Appendix 2).

A mother reported that her infant showed greater interest in various aspects of the natural world, such as birds and grass.

**Table 2:** Caregivers’ perceived effectiveness of the app.

Perceived effectiveness of the app	Example of feedback by caregivers	Participants, n^a^
Changes in caregivers’ awareness of time	- Began to think “Let my child go to bed earlier” - Began to be more aware of time in daily life	7
Changes in parenting behavior related to sleep habits	- Limiting nighttime media use - Creating positive bedtime routines preferred by their infants (eg, reading picture books) - Attempting to let their infant go to bed earlier	8
Improvement of infants’ sleep-related behavior	- “My child started going to bed more willingly and earlier than before” - “My child began to fall asleep sooner after going to bed”	4
Cessation of nighttime awakenings	- “My child has fewer nighttime awakenings than before”	2
Perceived reduction of childcare burden	- “It became more comfortable to take care of my infant since they sleep better than before” - “When my infant’s sleep got better, their behavior got better”	4

^a^Out of 10 participants.

## Discussion

### Main Findings

This pilot trial showed that there were no major problems with the system and the usability and acceptability of the app. High user dropout rates is one of the main issues of remote sleep interventions delivered via the internet and apps; results of one review study indicated a mean dropout rate as high as 78% for 5- to 6-week interventions [27]. These data showed that no participant dropped out during the 2 months of the trial. This might have occurred because (1) we included a convenience sample from a university community; (2) the study involved a reciprocal intervention, the encouragement of caregivers, and motivated users; (3) adequate sleep literacy via e-learning content contributed to motivating the caregivers; and (4) the app could have a better design in comparison with the previously tested ones. To better understand these issues, long-term interventions should be implemented in community-based trials.

Findings from the analysis of the reliability of the data entered by the caregivers were compatible with previous reports documenting the comparison of the sleep time recorded by actigraphy and the time recorded by parents [28-30].

The comparison between the actigraphy and the entered data showed that the latter were sufficiently reliable, particularly with respect to bedtime, which is also consistent with previous findings [28]. Regarding the discrepancies in the sleep duration and number of nighttime awakenings between the actigraphy data and the entered data, previous findings suggest that actigraphy could overestimate the number of awakenings depending on levels of body movement, especially in children whose body movement increases during sleep [30].

These results confirm that the modified actigraphy in this study resulted in findings similar to those of previous studies. Although it was not realistic that all the children wore the actigraphy device during the intervention, it is still a valuable data source for cases in which the caregiver report was judged to be unreliable. On the other hand, caregivers’ evaluations of the children’s bedtime and wake-up time were reliable in this study, as all the participants were educated and motivated. Taking this into account, the observed improvements in sleep habits, even in this short trial, are likely to reflect reality. The trial use of this app demonstrated the possible effectiveness of reciprocal interaction between caregivers and pediatric sleep experts. Furthermore, it could be effective in alleviating parenting burden among Japanese caregivers. As children’s bedtime refusal increases childcare burden for their caregivers, reductions in bedtime refusal are thought to alleviate the psychological and physical burden of caregiving on family life [31].

Although shorter sleep durations in Japanese children are well documented [9], few studies have focused on means to increase caregivers’ awareness and change parenting behavior effectively in Japan [32,33]. In addition, the factors that inhibit improvements in sleep literacy and changes in parenting behavior remain unclear. Although evidence demonstrating the importance of children’s sleep has accumulated, little attention has been paid to the circumstances and problems faced by caregivers who experience difficulty in improving their children’s sleep habits. Mindell et al [34] showed that lifestyle and living circumstances were related to sleep-related conditions in children. Allen et al [35] conceptualized the elements of adequate and good sleep in children in a review of studies examining sleep regularity, bedtime routines, quietness and noise comfort, lights, media use, activities, and family conflict. Fukumizu et al [36] suggested that co-sleeping habits and bedtime irregularity were associated with sleep-related nighttime crying in Japanese children. Changes in childcare practices that improve children’s sleep problems sometimes require considerable effort from caregivers, as many factors are associated with healthy sleep in children. For busy caregivers or caregivers who do not receive help with or appropriate advice regarding childcare, it is necessary to not only achieve appropriate levels of sleep literacy but also receive positive feedback regarding their efforts to change their behavior to improve children’s sleep habits. In this respect, the interactivity of the app functioned effectively.

From the rate at which caregivers attempted to follow advice (76.8%), we assume that sending advice to caregivers via the app was considered feasible.

The app could have contributed to changes in caregivers’ awareness of sleep and their parenting behavior using the PDCA cycle, throughout which they attempted to follow one piece of advice that they themselves chose. These changes in their awareness and parenting behavior could have led to changes in sleep habits and lifestyle (Multimedia Appendix 2). Although this was a short-term trial in which advice was provided only once, bathing time was earlier on weekends with the use of the app. As most of the caregivers who participated in this study worked on weekdays, changing their lifestyles on weekends may have been easier, as they had only one chance to get advice. Further research will include long-term trials that determine the effectiveness of this app in sleep habits on both weekdays and weekends. Future research may also measure caregivers’ awareness and behaviors that contribute to infants’ healthy sleep before and after the intervention.

### Study Strengths

This study shows that the app was successfully designed for reciprocal interaction between caregivers and pediatric sleep experts, which allowed all caregivers to receive personalized and appropriate information and advice. Additionally, the app allowed pediatricians to check caregivers’ intervention adherence.

### Limitations

This study was subject to some limitations. For example, as participation was voluntary and the participants were recruited from a university community, participants could have been healthier or possessed higher education levels relative to the general population, which could have led to selection bias. In addition, this study was conducted as a single-arm pilot study with a small sample because it aimed to check the usability and acceptability of the app. Furthermore, the intervention was implemented over a short period and it did not focus on the background factors of each family. Further studies should be conducted in consideration of these limitations.

### Conclusion

The results of this pilot study showed that the app developed for the intervention was acceptable and usable for Japanese caregivers. In addition, even though this was a preliminary trial, the app showed the scope of changes in children’s sleep habits, caregivers’ sleep health consciousness, and caregivers’ parenting behaviors in Japan. According to the users’ feedback, the caregivers welcomed the opportunity to obtain individualized advice that fit the backgrounds and home lives of each family based on the data participants entered via the app. This interactive system is the core feature of Nenne Navi.

Further studies need to be conducted to examine the app’s efficacy in improving sleep habits and its effects on follow-up maintenance in order to ensure social implementation of the app. Therefore, we will examine whether users maintain their motivation and high levels of compliance in long-term interventions in subsequent community-based trials. This app is expected to be used in sleep medicine and parental education in Japan and contribute to the expansion of sleep health literacy in families with infants. Moreover, the app could ultimately contribute to improvements in sleep habits and healthy development in Japanese children. Further research with a neuroscientific basis should be done to confirm whether this early sleep intervention leads to desirable brain development.

## Appendix

Multimedia Appendix 1 List of items for data entry via the app.

Multimedia Appendix 2 (A) Mean bedtime, (B) mean sleep duration, (C) mean time of finishing infant bathing.

## Abbreviations

PDCA: plan-do-check-act
