# Supplementary material for: An Interactive Smartphone App, Nenne Navi, for Improving Children’s Sleep: Pilot Usability Study
Source: JMIR Pediatr Parent. 2020 Dec 1;3(2):e22102. doi: 10.2196/22102 (PMC7738258; doi:10.2196/22102)
Supplement: Multimedia Appendix 2 [file pediatrics_v3i2e22102_app2.docx]

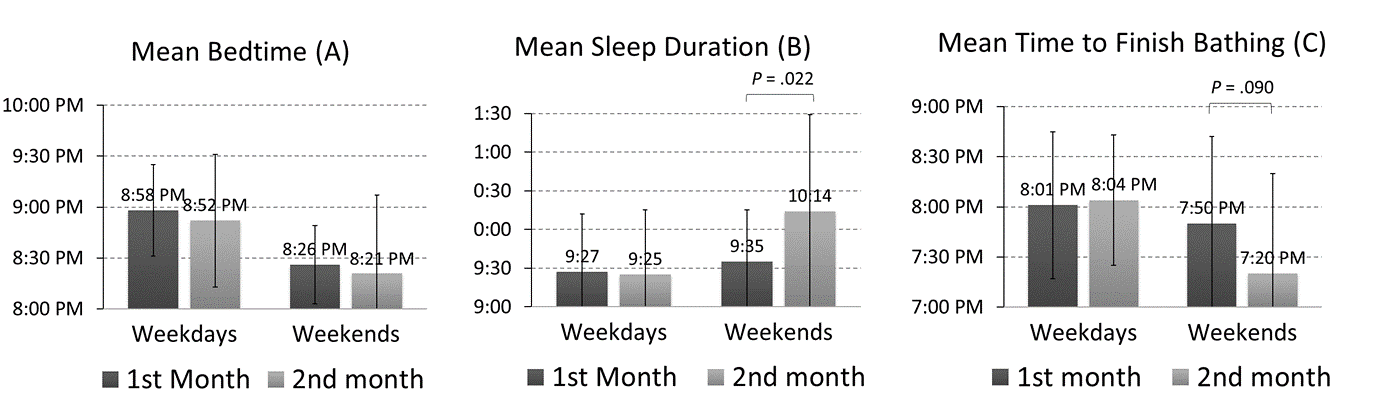


There was no significant difference in infants’ bedtimes between the first and second months (paired t-test, P = .388 for weekdays and P = .675 for weekends). The duration of infants’ sleep was longer in weekends (paired t-test, P = .022). In addition, there was a trend toward finishing bathing ahead of schedule on the weekends, but the result was non-significant (paired t-test, P = .090).
